# The Facility-Level HIV Treatment Cascade: Using a Population Health Tool in Health Care Facilities to End the Epidemic in New York State

**DOI:** 10.1093/ofid/ofy254

**Published:** 2018-10-26

**Authors:** Daniel J Ikeda, Leah Hollander, Susan Weigl, Steven V Sawicki, Daniel R Belanger, Nova Y West, Nanette Brey Magnani, Christopher G Wells, Peter Gordon, Johanne Morne, Bruce D Agins

**Affiliations:** 1 New York State Department of Health AIDS Institute, New York, New York; 2 HEALTHQUAL, Institute for Global Health Sciences, University of California, San Francisco, San Francisco, California; 3 Division of Infectious Diseases, Department of Medicine, Columbia University, New York, New York; 4 Institute for Implementation Science in Population Health, New York; 5 Graduate School of Public Health and Health Policy, City University of New York, New York

**Keywords:** HIV treatment cascade, quality of care, care engagement

## Abstract

**Background:**

The HIV treatment cascade is a tool for characterizing population-level gaps in HIV care, yet most adaptations of the cascade rely on surveillance data that are ill-suited to drive quality improvement (QI) activities at the facility level. We describe the adaptation of the cascade in health care organizations and report its use by HIV medical providers in New York State (NYS).

**Methods:**

As part of data submissions to the NYS Department of Health, sites that provide HIV medical care in NYS developed cascades using facility-generated data. Required elements included data addressing identification of people living with HIV (PLWH) receiving any service at the facility, linkage to HIV medical care, prescription of antiretroviral therapy (ART), and viral suppression (VS). Sites also submitted a methodology report summarizing how cascade data were collected and an improvement plan identifying care gaps.

**Results:**

Two hundred twenty-two sites submitted cascades documenting the quality of care delivered to HIV patients presenting for HIV- or non-HIV-related services during 2016. Of 101 341 PLWH presenting for any medical care, 75 106 were reported as active in HIV programs, whereas 21 509 had no known care status. Sites reported mean ART prescription and VS rates of 94% and 80%, respectively, and 60 distinct QI interventions.

**Conclusions:**

Submission of facility-level cascades provides data on care utilization among PLWH that cannot be assessed through traditional HIV surveillance efforts. Moreover, the facility-level cascade represents an effective tool for identifying care gaps, focusing data-driven improvement efforts, and engaging frontline health care providers to achieve epidemic control.

Ensuring that all persons living with HIV (PLWH) receive high-quality medical care remains a top priority in efforts to end the HIV/AIDS epidemic in the United States, yet the realization of this goal remains a challenge. In the absence of a foreseeable cure, suppression of plasma HIV RNA through antiretroviral therapy (ART) continues to be the desired outcome of effective care for HIV infection, conferring both individual and public health benefits. However, despite the wide availability of ART in the United States, viral suppression (VS) is achieved by only a fraction of PLWH amid widening racial and socioeconomic disparities [1, 2].

In efforts to better understand why low rates of VS persist, research continues to document the individual- and structural-level correlates of VS. The HIV treatment cascade, a visual representation of the sequential steps between HIV diagnosis and VS, constitutes a powerful framework within which to understand these correlates of VS at the population level and prioritize areas for improvement [3, 4]. For example, although only 70% of diagnosed PLWH in New York State (NYS) were estimated to be virally suppressed at the end of 2016, the rate of VS among diagnosed PLWH with any care (defined as evidence of viral load, CD4, or genotype test in the previous year) was 87% [5], confirming that improvement of care engagement among PLWH represents a key strategy for improving rates of VS and preventing onward transmission [5, 6].

Despite the utility of the HIV treatment cascade in characterizing population-level gaps in HIV care outcomes, its suitability for driving quality improvement (QI) activities at the facility level is limited. A key drawback of population-level HIV quality metrics, for example, is the way in which “engagement” is defined. Indeed, an unpublished analysis of Medicaid claims data by the New York State Department of Health (NYSDOH) in 2015 found that many PLWH without evidence of HIV care engagement were utilizing non-HIV-related care services in health care organizations with HIV clinics, including emergency departments (EDs) and mental health and dental services. For feedback of clinical performance data to be effective in spurring QI efforts, these data must be perceived as timely, credible, customizable, and contextually meaningful by clinicians [7–9]. Unfortunately, because national, regional, state, and city HIV treatment cascades rely on population-level surveillance data for assessing “engagement,” they are often too untimely—due to months-long lags in reporting—to evaluate the success of QI activities in real time. Moreover, as these data are routinely aggregated above the site level, clinical staff are unable to apply QI interventions to their local contexts and unique patient populations.

In light of these key limitations, the NYSDOH AIDS Institute adapted the construct of the HIV treatment cascade to the facility level, embracing a public health approach to QI that directly links site-level activities to jurisdictional cascades. In pursuit of this objective, the facility-level cascade framework was developed to equip sites with a standardized tool to (1) monitor the extent and quality of care being delivered to all PLWH seen at their facility, not only those enrolled in their HIV medical programs; (2) engage facility providers in the full sequence of steps in the treatment cascade for patients receiving any type of care in their facilities; (3) identify gaps in the sequences of steps between diagnosis and VS as they are delineated by the cascade; and (4) develop data-driven plans to assess and improve these gaps through QI activities. In this investigation, we report the implementation of the facility-level cascade in NYS health care facilities and describe its integration into a coordinated policy strategy to achieve statewide epidemic control.

## METHODS

### Policy Context

In 2014, Governor Andrew M. Cuomo launched the Ending the AIDS Epidemic Initiative, a 3-point plan to reduce the number of annual new infections in NYS to below 750 by 2020 [10]. In alignment with these activities, facilities that provide HIV care in NYS were instructed to develop facility-level HIV treatment cascades as part of required data submissions to the NYS HIV Quality of Care Program of the NYSDOH AIDS Institute. Launched in 1992 and administered with guidance from consumer and provider advisory committees, the NYS HIV Quality of Care Program is responsible for the systematic monitoring of HIV processes and outcomes in NYS and applies QI methods to the achievement of desired clinical outcomes for PLWH [11]. The full scope of the program’s activities has been described elsewhere [12].

### Submission Requirements

In November 2016, a guidance document that outlined the required elements for submission was disseminated to facilities providing HIV medical care in NYS. In the guidance document, facilities were asked to submit HIV treatment cascades that captured a suite of required measures for *all* PLWH who received *any* services between January 1, 2016, and December 31, 2016, regardless of whether these patients were formally enrolled in the facilities’ HIV programs. This approach contrasts with previous NYS requirements that did not consider health care utilization of PLWH outside of HIV programs. To facilitate presentation and interpretation of data and to target improvement efforts, facilities were instructed to construct 2 separate cascades: 1 for newly diagnosed patients and 1 for previously diagnosed patients. In addition to submission of 2 cascades, facilities were asked to submit a formal methodology report summarizing how cascade data were collected and analyzed and an action plan for improving gaps in care identified through facilities’ interpretations of their cascades.

### Measures

In the HIV treatment cascade for newly diagnosed patients (ie, those diagnosed during the measurement year), sites were required to capture data on 3 measures: (1) linkage to HIV medical care, (2) ART prescription, and (3) VS (Table 1). In contrast to the standard surveillance definition, which specifies successful linkage to HIV medical care as documented receipt of a viral load, CD4, or genotype test within 30 days of diagnosis [13], in the facility-level cascade, successful linkage was defined as attendance at an appointment with an HIV provider within 3 days if referred within the facility, or within 5 days if referred to an outside facility. With formal approval by the NYS HIV Quality of Care Clinical Advisory Committee, this definition was adopted in response to increasing evidence supporting the efficacy of same-day ART initiation in accelerating time to VS in newly diagnosed patients [14–16]. In the HIV treatment cascade for previously diagnosed patients (ie, those diagnosed before the measurement year), sites were required to report data on 4 measures: (1) open patient caseload, (2) active patient caseload, (3) ART prescription, and (4) VS (Table 1). In light of findings demonstrating a weak association between retention in care (24-month visit constancy measure) and VS among PLWH in NYS [17], longitudinal retention in HIV medical care was not included as a required measure. However, if sites determined retention or other clinical performance indicators to be worthwhile to track their patient population, then they were encouraged, but not required, to include them as measures in their cascade submissions. All measures were defined according to NYS HIV Clinical Care Guidelines, with the exception of linkage to care, which is not formally defined therein.

**Table 1.** **Required Measures: Newly Diagnosed and Previously Diagnosed Patient Cascades**

Newly Diagnosed Patient Cascade	
Measure	Description
Newly diagnosed caseload Linkage to HIV medical care	The number of PLWH diagnosed with HIV at the facility The proportion of newly diagnosed patients who attended an appointment with an HIV provider within 3 days if referred within the facility, or within 5 days if referred outside the facility
ART prescription	The proportion of newly diagnosed patients who had an active prescription for ART at the end of the calendar year
Viral suppression	The proportion of newly diagnosed patients who were virally suppressed (<200 copies/mL) at the last viral load test of the calendar year
Previously Diagnosed Patient Cascade	
Measure	Description
Open patient caseload	The number of PLWH receiving any medical service within the facility, regardless of whether they were formally enrolled in its HIV program
Active patient caseload	The proportion of open caseload patients who received services in the HIV program during the calendar year
ART prescription	The proportion of active caseload patients who had an active prescription for ART at the end of the calendar year
Viral suppression	The proportion of active patients who were virally suppressed (<200 copies/mL) at the last viral load test of the calendar year

Abbreviations: ART, antiretroviral therapy; PLWH, people living with HIV.

### Technical Support and Coaching

After the release of the guidance document in November 2016, facilities were assigned an improvement coach from the NYS HIV Quality of Care Program with whom to address concerns and troubleshoot challenges. In addition, Quality of Care Program staff hosted a series of weekly webinars that provided a step-by-step summary of the guidance document and answered frequently asked questions that emerged during coaching activities. Beginning in January 2017 and in advance of the submission deadline of March 31, 2017, sites with completed submissions were invited to share their results, best practices, and proposed improvement interventions during these weekly webinars and as part of existing QI learning network activities.

### Evaluation and Approval

Following the deadline for submission, sites’ cascade submissions underwent a 4-step evaluation process. In the first step, submissions were screened for required components. In the second step, sites’ QI plans were examined by their assigned improvement coach. In step 3, sites’ methodology reports were reviewed by a member of the Quality of Care Program. In the fourth and final step, the entire submission was reviewed in light of the previous reviewers’ comments by the Medical Director of the NYSDOH AIDS Institute, and it was either approved or rejected with a request for revision or clarification.

## RESULTS

**Figure 1.**
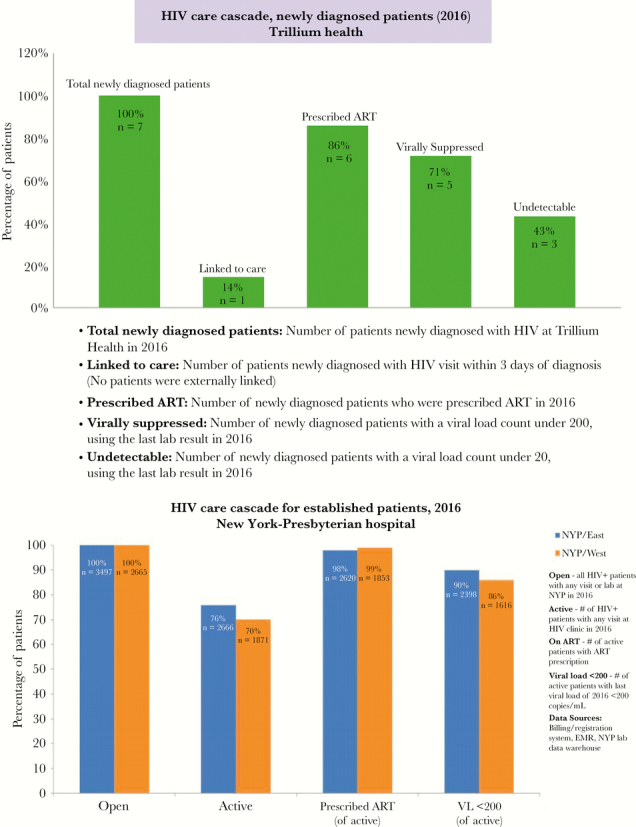
Example facility-level cascades. Abbreviations: ART, antiretroviral therapy; EMR, electronic medical record; NYP, New York-Presbyterian; VL, viral load.

Two hundred twenty-two facilities spanning 81 organizations submitted HIV treatment cascades. Among submitting facilities, 57% were classified as community health centers, 30% as hospitals, and 13% as drug treatment programs; 65% were located in New York City. Cascade submissions varied considerably in approach, with some facilities using multiple data sources to construct their cascades. In addition, submissions varied significantly in terms of completeness, with some requiring multiple rounds of revisions and intensive coaching. Challenges that were commonly encountered by sites in cascade construction included data missingness, delayed involvement of information technology personnel to query facility-wide data systems for identification of open patients, difficulties reconciling multiple data sources, and time costs associated with gathering and merging data contained in unstructured electronic medical record fields. Identified gaps commonly reported by sites in their improvement plans included suboptimal documentation of the care status of open caseload patients (particularly those receiving care in emergency departments), disparities in VS by key population, and suboptimal linkage to care rates. An example cascade for newly diagnosed patients from Trillium Health, a community health center in Rochester, New York, and an example cascade for previously diagnosed patients from New York-Presbyterian Hospital, a tertiary facility in New York, New York, are displayed in Figure 1.

### Performance Measurement

Performance measurement data contained in submitted cascades were reviewed by program staff and collated (Table 2). Because submission of unique identifiers was not required as part of cascade submissions—given the initiative’s primary aim to describe and act upon data specific to a given facility—de-duplication of patients across organizations was not possible; accordingly, all reported data are unweighted. Submitting organizations reported a total of 101 341 PLWH who received any services in 2016, of whom 75 106 (74%) were identified as being actively enrolled in their HIV programs. Of 26 235 patients who received services (“open”) but did not receive HIV-related medical services (“active”), 21 509 (82%) were ascribed an unknown care status, meaning they could not be confirmed to be deceased, incarcerated, or enrolled in HIV medical care at another organization. The number of patients with unknown care status was highly variable across organizations (range, 0–4025; interquartile range, 3–196). Average reported ART prescription rates among active previously diagnosed patients were high among submitting organizations (mean, 94%). Rates of VS among active previously diagnosed patients were comparable with those from the statewide cascade after adjustment to include only PLWH with any evidence of care (mean, 80%). Organizations reported 1777 patients as newly diagnosed with HIV in 2016. The mean reported linkage to care rate for newly diagnosed patients was 52%, and the mean reported ART prescription rate was 76%. Among submitting facilities, the mean reported VS rate at last viral load test (unadjusted for time on ART) was 55%.

**Table 2.** Summary Performance Measurement Data, All Organizations

Patients by Care Status	
Care status	No. (IQR)
Newly diagnosed patients	1777 (4–24)
Previously diagnosed open patients	101 367 (217–1056)
Previously diagnosed active patients	75 109 (142–795)
Previously diagnosed patients with unknown care status	21 517 (3–196)
Previously diagnosed patient cascade measures	
Measure	Mean (IQR), %
ART prescription	94 (93–99)
Viral suppression	80 (75–89)
Newly diagnosed patient cascade measures	
Measure	Mean (IQR), %
Linkage to care	52 (14–97)
ART prescription	76 (60–100)
Viral suppression	55 (36–70)

Abbreviations: ART, antiretroviral therapy; IQR, interquartile range.

### Improvement Interventions

As part of cascade submissions, facilities were instructed to identify action steps or interventions for areas of the cascade where performance was shown to be suboptimal. Facilities selected interventions in different ways, ranging from team-based problem solving and root cause analysis to adaptation of evidence-based solutions identified in the literature or successfully implemented by other organizations. Following submission, the changes adopted by sites were then grouped by staff according to the concepts of the intervention, such as reminders or patient involvement in care planning, which are presented in Table 3 according to domains based on the expanded Chronic Care Model [18–20], with adaptation to the preventive medicine and public health context [21]. Specifically, the domain of self-management was broadened to include all elements of patient-centered care, decision support was expanded to include knowledge management strategies, and information systems was integrated into the broader domain of performance measurement. Finally, a category of financial interventions was added. In total, facilities reported 60 distinct interventions across these domains.

**Table 3.** Interventions Adopted by Sites, by Chronic Care Model Domain

Chronic Care Model Domain	Interventions
Health system	• Integration of cascade into existing HIV quality management plan and program • Interprofessional team rounds • Referrals and service programs and agencies • Expanded clinic hours • Inclusion of community health workers and peers in case management and QI teams • Coordination with other service delivery areas and departments to identify and refer PLWH to HIV care • Implementation of memoranda of understanding with other agencies to share information and establish care referral policies
Delivery system	• Rearrangement of clinic flow • Spacing of clinic visits based on need • Home visits • Care navigation for clinic appointments • Intensified screening for mental health and substance use • Reminder strategies • Flexible appointment scheduling for new and unengaged patients • Telemedicine and e-visits • Adherence counseling at first clinic visit • Individualized care plans for ART initiation • HIV lab testing for patients receiving care in other service delivery areas • Directly observed therapy • Transition plans for adolescent patients transferring to adult care
Patient-centered care	• Patient involvement in care planning and case conferencing • Shared decision-making • Involvement of consumer advisory groups to identify effective interventions and participate in QI activities • Implementation and use of online patient portals • Use of visuals and videos to address health literacy • Use of adherence tools • Implementation of self-management programming • Motivational interviewing • Peer support groups • Personal cascade narratives • Use of social media to communicate adherence promotion strategies
Knowledge management and decision support	• Education of HIV program staff on cascade methodology • Sensitization of staff in other service delivery areas and departments about unengaged PLWH • Data transparency policies • Education of HIV program staff about HIV-related stigma • Referral resources guide • Formal policy and training on same-day ART initiation • Training of HIV program staff on entitlement programs • Education of providers about refill standards
Information systems and performance measurement	• Updated patient contact information • Frequent and automated report generation to track virally unsuppressed patients • Previsit patient reports for care coordination planning • Structured templates in EMRs • Structured fields in EMRs for care coordination • Monitoring of prescription refill rates • Daily alert system with updates on new patients and test results • Tracking time from diagnosis to first clinic visit • Monitoring of staff compliance to linkage-to-care policies • Routine reporting of missed appointments and labs within specified interval • Provision of tracking information to case management team • Integration of visit tracking systems into EMRs
Community	• Engagement of community partners to promote linkage and VLS • Linkage of patients to community services • Inclusion of community partners in care planning • Referral of LTFU patients to health department • Routinized communication with community partners to confirm linkage to care • Partnership with insurance companies to facilitate care enrollment • Linkage to transportation services • Outreach community groups catering to specific at-risk subpopulations
Financial	• Incentives for retention and viral load suppression

Abbreviations: ART, antiretroviral therapy; EMR, electronic medical record; LTFU, lost to follow-up; PLWH, people living with HIV; QI, quality improvement; VLS, viral load suppression.

## DISCUSSION

The HIV treatment cascade has been widely used to present population-level data on care engagement from diagnosis through VS. However, as common adaptations of the cascade are constructed using surveillance data, their utility in spurring targeted and timely QI interventions at the facility level to address disengaged patients is limited. In this paper, we have proposed a new adaptation of the cascade that uses facility-generated data to drive point-of-care improvement efforts, and we have demonstrated the feasibility of its implementation as a statewide HIV QI initiative. To our knowledge, the NYS facility-level cascade initiative is the first systematic effort to encourage providers to reach beyond their HIV medical programs to ensure the care engagement of all PLWH—and not simply those formally enrolled in their care.

Engagement of PLWH in medical care that facilitates the achievement of VS remains a key challenge in efforts to end the HIV/AIDS epidemic in NYS and the United States. In response to the pressing need to return disengaged PLWH to care, state and city health departments have begun to adopt “data-to-care” initiatives—a public health strategy recommended by the Centers for Disease Control and Prevention in which HIV surveillance data are used to identify PLWH without evidence of HIV care and target them through community outreach for linkage or re-engagement [22–25]. To complement this strategy, the facility-level cascade equips providers with timely, locally generated data to identify PLWH at the point of care, ascertain their care status, and attempt to relink those found to be disengaged.

That more than 20 000 PLWH presenting for non-HIV-related services in NYS had no documented care status in clinical records is a troubling finding of the current work, and highlights a clear shortcoming in full adoption of a public health approach to ending the epidemic. This challenge of care status ascertainment was particularly acute in EDs and is of particular concern given their role as primary points of entry into health care organizations for sporadically engaged PLWH seeking medical services [26]. In addition to EDs [27], other health care safety net institutions such as public sexual health clinics have reported similar challenges in ascertaining care status [28]. Based on our available data, we cannot precisely differentiate PLWH of “unknown care status,” whose providers failed to ask about their care status, from those whose care status was ascertained but simply entered into an unstructured electronic medical record (EMR) field and thus not reported. Both constitute plausible explanations for the high reported prevalence of unknown care status and underscore the need to ensure that the care status of all PLWH is not only ascertained, but easily queried to target interventions in real time.

Although health care organizations have developed HIV-specific registries for the purposes of mandatory reporting and may use them as part of QI activities, these data systems are generally confined to their HIV programs, with varying levels of integration with other clinical data systems within the larger institution. Inter- and intra-organizational boundaries—especially in IT systems—routinely lead to discontinuous coordination of care and constitute a well-documented barrier to QI implementation [29]. In recent years, the reach of regional health information organizations (RHIOs) and other health information exchanges has expanded in NYS and elsewhere, enabling providers to track the care status and outcomes of PLWH both within and across participating institutions. However, despite evidence of their utility in the context of HIV care [30, 31], the uptake of RHIOs has been disappointingly slow [32], signifying a missed opportunity for “meaningful use” of these systems to improve the coordination of care for PLWH. As adoption of these systems has long been viewed as a means to achieve interoperability across the US health system, further work is needed to understand not only how to incentivize data sharing across organizations, but how to ensure that these data can be used at the point of care—through interventions such as incorporation of structured EMR fields to encourage routine ascertainment of care status and real-time notifications of care utilization [33]—in order to re-engage out-of-care PLWH.

The current work has limitations. First, because all data contained in cascade submissions were self-reported by sites and influenced by organizational idiosyncrasies in data collection approach and quality, it is unclear whether these data are fully consistent with estimates presented in state-level surveillance reporting. Second, as unique identifiers were not reported as part of cascade submissions, de-duplication of patients across organizations was not feasible. Accordingly, the precise number of “open” patients cannot be ascertained. Third, as this study documents the first year of this statewide initiative, it cannot assess whether implementation of facility-level cascades—and associated interventions—was associated with site-level improvement in indicator performance. Future work is warranted to explore the magnitude of these improvements and the utility of cascade data in generating and evaluating efficacious interventions.

## CONCLUSIONS

In support of NYS’s Ending the Epidemic Initiative, which envisions the end of the AIDS epidemic in NYS by 2020, concerted efforts are needed to identify out-of-care PLWH and relink them to HIV medical care. This study documents the adaptation of the HIV treatment cascade to the facility level as part of routine QI activities. Because traditional QI approaches are limited in reach to only PLWH formally enrolled in HIV medical programs—as opposed to all PLWH presenting for medical services—facility-level HIV QI activities often neglect care engagement of PLWH within their own organizations, and consequently fail to address the full spectrum of HIV care and treatment. The visual display of the facility-level cascade represents a novel strategy to engage providers across inter- and intra-organizational boundaries to leverage locally generated data to achieve epidemic control.

## Notes

***Prior presentation:*** Parts of the data in this manuscript were presented at the 12th International Conference on HIV Treatment and Prevention Adherence; June 4–6, 2017; Miami, FL.
